# First Complete Genome Sequence of Yersinia massiliensis

**DOI:** 10.1128/genomeA.00416-18

**Published:** 2018-05-17

**Authors:** Matthew C. Thomas, Victoria Arling, Noriko Goji, Timothy W. Janzen, Marc-Olivier Duceppe, Amit Mathews, Catherine Carrillo, Kingsley K. Amoako

**Affiliations:** a Calgary Laboratory, Canadian Food Inspection Agency, Calgary, Alberta, Canada; b Lethbridge Laboratory, Canadian Food Inspection Agency, Lethbridge, Alberta, Canada; c Ottawa Laboratory Fallowfield, Canadian Food Inspection Agency, Ottawa, Ontario, Canada; d Greater Toronto Area Laboratory, Canadian Food Inspection Agency, Scarborough, Ontario, Canada; e Ottawa Lab Carling, Canadian Food Inspection Agency, Ottawa, Ontario, Canada

## Abstract

Using a combination of Illumina paired-end sequencing, Pacific Biosciences RS II sequencing, and OpGen Argus whole-genome optical mapping, we report here the first complete genome sequence of Yersinia massiliensis. The completed genome consists of a 4.99-Mb chromosome, a 121-kb megaplasmid, and a 57-kb plasmid.

## GENOME ANNOUNCEMENT

Yersinia massiliensis is a recently recognized bacterial species (1, 2) that includes yersinial strains previously classified as Y. frederiksenii genomospecies 2 (3). Y. massiliensis is a Gram-negative coccobacillus and is considered to be nonpathogenic (4). The type strain, Y. massiliensis CCUG 53443, was isolated circa 2008 from freshwater in Marseilles, France (1). Here, we present the first complete genome of Y. massiliensis strain GTA, which was isolated from dried tofu as part of routine sampling at the Canadian Food Inspection Agency.

DNA for single-molecule real-time (SMRT) sequencing was prepared using the Genomic Tip 20/G kit (Qiagen, Hilden, Germany) per the manufacturer’s instructions, and sequencing was performed using 1 SMRT cell in a PacBio RS II sequencer (Pacific Biosciences, Menlo Park, CA, USA) at the Innovation Centre at McGill University (Genome Québec, Montréal, QC, Canada). DNA for MiSeq sequencing was extracted with the Epicentre MasterPure Gram-positive DNA purification kit (Illumina, San Diego, CA, USA). MiSeq libraries were prepared from 1 ng of genomic DNA using the Nextera XT kit, and sequencing was completed with the MiSeq V3 (600-cycle) reagent kit (Illumina). DNA for whole-genome optical mapping was extracted with the Argus HMW DNA isolation kit (OpGen, Gaithersburg, MD, USA) per the manufacturer’s instructions and digested with AflII to generate ordered restriction maps on the Argus optical mapping system (OpGen).

PacBio SMRT sequencing yielded 98,682 polymerase reads with a mean length of 11,767 bp and 1,161,211,588 sequenced bases, which were assembled *de novo* using the Hierarchical Genome Assembly Process (HGAP) workflow (5). The assembly generated five contigs with lengths of 5 Gb, 143 Mb, 24 Mb, 1.2 Mb, and 564 bp and with average coverages of 182×, 215×, 21×, 9×, and 2×, respectively. Illumina paired-end sequencing yielded 4,509,794 reads, which were error corrected using BBNorm and merged with BBMerge version 35.82 (6). Merged reads were used in conjunction with PacBio contigs as input for Circlator version 1.51 (7), which circularized the largest two contigs. A hybrid assembly of Illumina paired-end reads and PacBio contigs was performed with SPAdes version 3.11.1 (8, 9) using the “–careful” and “–trusted-contigs” flags. The assembly yielded contigs of 4.99 Mb, 122 kb, and 60 kb; the uncircularized PacBio contigs mapped to the 60-kb contig, which was then circularized with Circlator. Error-corrected paired-end MiSeq reads were then mapped to the closed chromosome and plasmids using Bowtie 2 version 2.3.3.1 (10), which yielded average coverages of 212× for the chromosome, 373× for the 122-kb plasmid, and 167× for the 60-kb plasmid. Mapped reads were used as input for Pilon version 1.22 (11) to polish the assembly. The polished closed genome was imported into MapSolver version 3.2.4 (OpGen) and compared to the optical maps to confirm the assembly. Features of the completed genome were annotated with the NCBI Prokaryotic Genome Annotation Pipeline (12).

The complete Y. massiliensis strain GTA genome consists of a 4,986,001-bp chromosome, a 121,622-bp megaplasmid, and a 57,277-bp plasmid with GC contents of 47.7, 46.3, and 40.1%, and 4,401, 115, and 64 predicted genes, respectively. No acquired antimicrobial resistance genes were detected with ResFinder (13).

### Accession number(s).

The complete genome sequence of Yersinia massiliensis strain GTA has been made available in NCBI GenBank under accession numbers CP028487 to CP028489.
